# Comprehensive genomic analysis of the SARS-CoV-2 Omicron variant BA.2.76 in Jining City, China, 2022

**DOI:** 10.1186/s12864-024-10246-w

**Published:** 2024-04-17

**Authors:** Qiang Yin, Wei Liu, Yajuan Jiang, Qiang Feng, Xiaoyu Wang, Huixin Dou, Zanzan Liu, Feifei He, Yingying Fan, Baihai Jiao, Boyan Jiao

**Affiliations:** 1 Department of Laboratory, Jining Center for Disease Control and Prevention, Jining, China; 2 Department of Laboratory, Rencheng Center for Disease Control and Prevention, Jining, China; 3 Computer Information Technology, Northern Arizona University, Arizona, USA; 4 Department of Medicine, School of Medicine, University of Connecticut Health Center, Farmington, CT USA

**Keywords:** SARS-CoV-2, Omicron, Whole genome sequencing, Molecular features, Termination mutation

## Abstract

**Objective:**

This study aims to analyze the molecular characteristics of the novel coronavirus (SARS-CoV-2) Omicron variant BA.2.76 in Jining City, China.

**Methods:**

Whole-genome sequencing was performed on 87 cases of SARS-CoV-2 infection. Evolutionary trees were constructed using bioinformatics software to analyze sequence homology, variant sites, N-glycosylation sites, and phosphorylation sites.

**Results:**

All 87 SARS-CoV-2 whole-genome sequences were classified under the evolutionary branch of the Omicron variant BA.2.76. Their similarity to the reference strain Wuhan-Hu-1 ranged from 99.72 to 99.74%. In comparison to the reference strain Wuhan-Hu-1, the 87 sequences exhibited 77–84 nucleotide differences and 27 nucleotide deletions. A total of 69 amino acid variant sites, 9 amino acid deletions, and 1 stop codon mutation were identified across 18 proteins. Among them, the spike (S) protein exhibited the highest number of variant sites, and the ORF8 protein showed a Q27 stop mutation. Multiple proteins displayed variations in glycosylation and phosphorylation sites.

**Conclusion:**

SARS-CoV-2 continues to evolve, giving rise to new strains with enhanced transmission, stronger immune evasion capabilities, and reduced pathogenicity. The application of high-throughput sequencing technologies in the epidemic prevention and control of COVID-19 provides crucial insights into the evolutionary and variant characteristics of the virus at the genomic level, thereby holding significant implications for the prevention and control of the COVID-19 pandemic.

**Supplementary Information:**

The online version contains supplementary material available at 10.1186/s12864-024-10246-w.

The Coronavirus Disease 2019 (COVID-19) is an acute respiratory infectious disease caused by the novel coronavirus, severe acute respiratory syndrome coronavirus 2 (SARS-CoV-2) [1]. Since its outbreak in December 2019, it has posed a serious threat to global public health [2, 3]. SARS-CoV-2, a single-stranded positive-sense RNA virus, exhibits a high mutation rate, leading to the emergence of multiple variants of concern (VOC) as identified by the World Health Organization, including the Alpha variant, Beta variant, Gamma variant, Delta variant, and the Omicron variant. The Omicron variant, first detected in Africa in November 2021, has since become the dominant strain worldwide due to its significant mutations, contributing to its increased transmissibility [4, 5].

With the Omicron variant’s continuous evolution, several subvariants have emerged, namely BA.1, BA.2, BA.3, BA.4, and BA.5 [6, 7]. In August 2022, the BA.2.76 subvariant was identified in Liaoning Province, China, marking a new phase in the virus’s adaptation. This subvariant’s spread to other regions such as Sichuan, Chongqing, Hebei, and Gansu have underscored the ongoing challenges in managing the COVID-19 epidemic within China. T Against this backdrop, our study focused on the Omicron BA.2.76 subvariant, conducting whole-genome sequencing and genetic evolution analysis on specimens from 87 cases in Jining City in 2022. This investigation aims to deepen our understanding of the SARS-CoV-2’s evolutionary dynamics and variant characteristics, thereby enhancing the data available for COVID-19 prevention and control strategies in China.

The emergence of the Omicron variant, particularly the BA.2.76 subvariant, underscores the need for a multifaceted response encompassing vigilant surveillance, continuous research, and adaptive public health strategies. Initial observations suggest that while the Omicron variant demonstrates enhanced transmissibility, its impact on disease severity and the effectiveness of existing vaccines may vary, necessitating ongoing evaluation and possible adjustments to vaccination policies. This context of rapid viral evolution and the emergence of new subvariants highlights the critical importance of localized genomic studies, such as ours, in contributing to the global understanding of COVID-19’s trajectory and informing tailored response measures.

## Materials and methods

### Specimen collection

From September 1, 2022, to October 4, 2022, throat swab specimens from suspected cases of early or community-detected Omicron variant BA.2.76 outbreak infections were collected in Jining City, Shandong Province, China. The swab samples were placed in virus sampling tubes (Jinan Biobio Biotechnology Co., Ltd.), kept at 2–8℃, and transported to the microbiology laboratories of the Jining City Center for Disease Control and Prevention and/or the microbiology laboratories of the Rencheng District Center for Disease Control and Prevention within 2 h.

### Nucleic acid detection

Throat swab specimens (200 µL) were obtained and underwent nucleic acid extraction using the qEx-DNA/RNA Virus Nucleic Acid Extraction or Purification Kit (Xi’an Tianlong Technology Co., Ltd.) and the Libex 96 Fully Automatic Nucleic Acid Extractor (Xi’an Tianlong Technology Co., Ltd.). Nucleic acid testing was conducted using the Coronavirus 2019-nCoV Nucleic Acid Detection Kit (Real-time Fluorescent Quantitative PCR) from Shanghai Bio-Germ Medical Technology Co., Ltd. and the Gentier Real-time Fluorescent Quantitative PCR Instrument (Xi’an Tianlong Technology Co., Ltd.).

### Genetic sequencing

For the comprehensive analysis of SARS-CoV-2 nucleic acid in positive specimens, we employed the ULSEN ultra-sensitive coronavirus whole-genome capture kit (Beijing Weimai Future Technology Co., Ltd). This sophisticated kit facilitated the targeted capture and amplification of the entire SARS-CoV-2 genome. Subsequent to amplification, the resultant products underwent purification using the AMpure XP nucleic acid purification kit, also from Beijing Weimai Future Technology Co., Ltd. Library construction followed, leveraging the Nextera XT DNA Library Preparation kit and Nextera XT Index Kit v2 Set A, (Illumina, Inc. (USA)). Post-library preparation, the sequencing of the SARS-CoV-2 whole genome was executed utilizing the MiSeq Reagent Kit v2, 300-cycles, and the MiSeq sequencer, manufactured by (Illumina, Inc.). The data presented in the study are deposited in the National Microbiology Data Center (NMDC), accession number list in Supplementary Table S1.

### Bioinformatics analysis

Sequencing data from the MiSeq instrument underwent assembly of the SARS-CoV-2 genome using CLC Genomics Workbench 21 by QIAGEN. Variant analysis was performed through Nextclade v2.14.1 (https://clades.nextstrain.org/). Reference (Wuhan-Hu-1, GenBank: MN908947.3) and variant sequences were obtained from NCBI and GISAID. Nucleotides 67-29703 of the SARS-CoV-2 whole genome sequence were selected for alignment using the Alignment By Muscle feature in MEGA 7.0.14. The SARS-CoV-2 evolutionary tree was constructed using MEGA 7.0.14, employing the maximum-likelihood method with 1000 bootstrap replicates. Further analyses included amino acid mutation identification, 5’UTR and 3’UTR structure determination with MEGA 7.0.14 and RNAfold, respectively. N-glycosylation sites were analyzed using NetNGlyc-1.0 (https://services.healthtech.dtu.dk/services/NetNGlyc-1.0/), and phosphorylation sites were assessed via NetPhos-3.1 (https://services.healthtech.dtu.dk/services/NetPhos-3.1/).

## Results

### Whole genome sequencing analysis

**Fig. 1:**
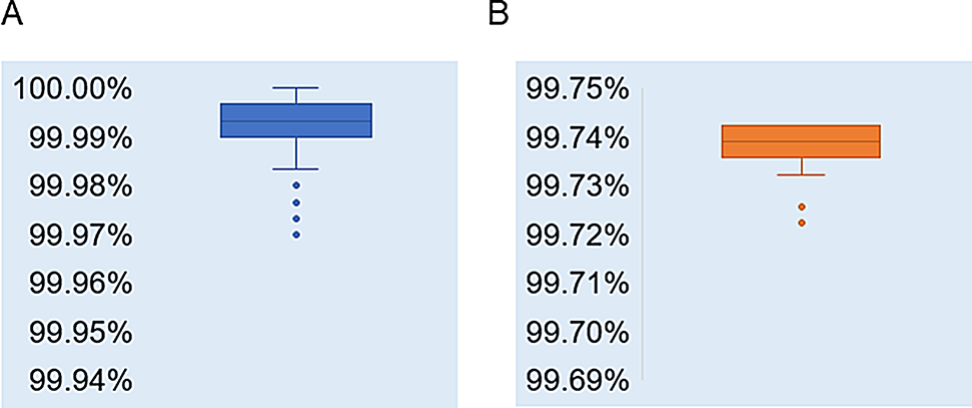
.

From September 1 to October 4, 2022, COVID-19 outbreak occurred in Jining City, Shandong Province, China. Whole genome sequencing of SARS-CoV-2 was conducted on specimens collected from infected individuals during this period, resulting in 87 complete SARS-CoV-2 genome sequences. The sequences ranged in length from 29,669 bp to 29,860 bp, and were designated as hCoV-19/Jining/JN01/2022 - hCoV-19/Jining/JN87/2022. The sequence coverage ranged from 99.2 to 99.9%. Analysis using Nextclade v 2.14.1 confirmed that all sequences belong to the Omicron BA.2.76 variant. The nucleotide sequence similarity among the 87 sequences ranged from 99.97 to 100% (Fig. 1A). When compared to the Wuhan-Hu-1 reference sequence, the similarity of the 87 sequences ranged from 99.72 to 99.74% (Fig. 1B).

### Genetic evolution analysis

**Fig. 2:**
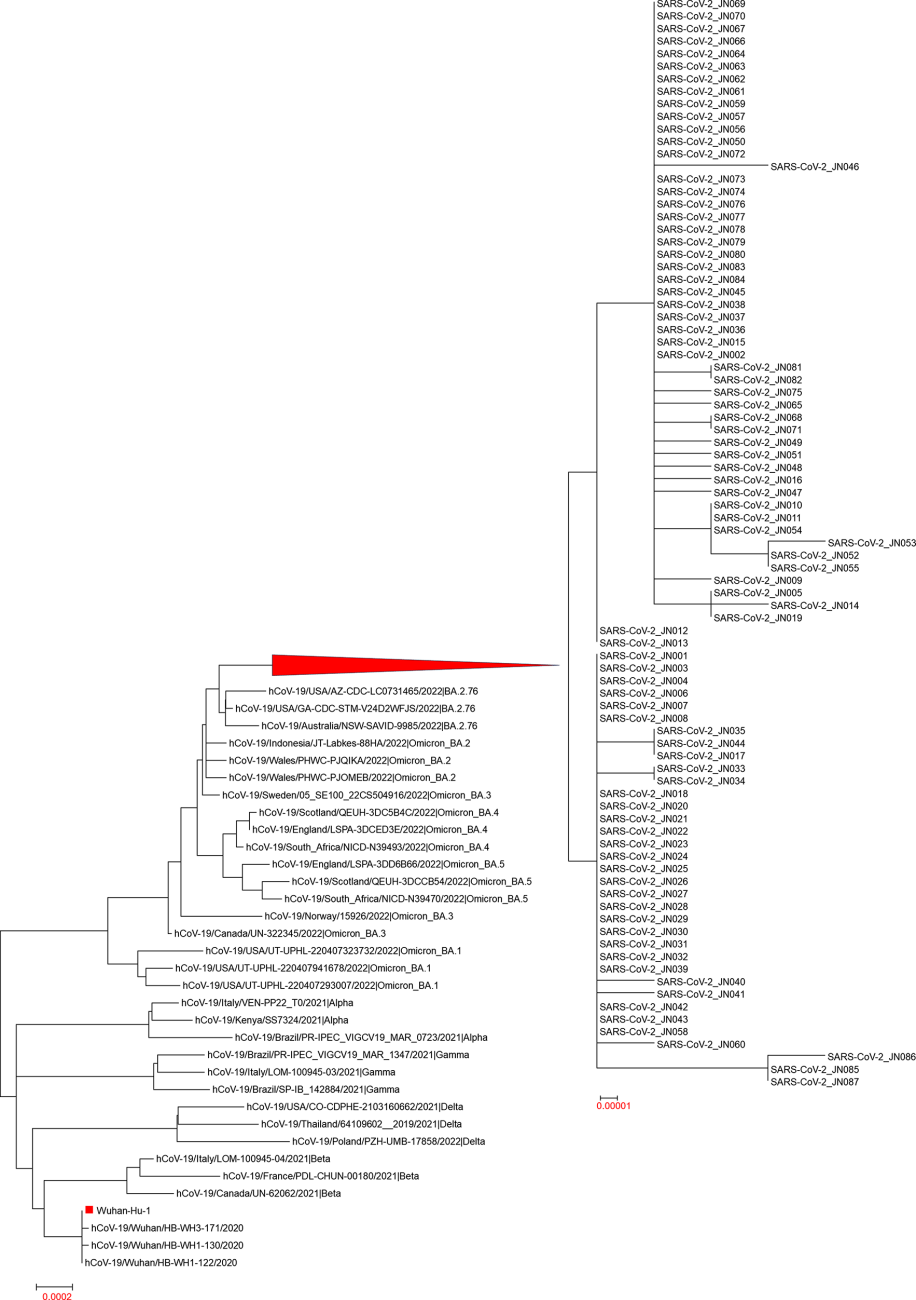
.

Utilizing 87 Omicron BA.2.76 sequences obtained from Jining City, we conducted a comprehensive genetic evolution analysis by constructing an evolutionary tree. This tree included reference sequences such as Wuhan-Hu-1, early isolates of the novel coronavirus, and variants like Alpha, Beta, Gamma, Delta, Omicron BA.1, Omicron BA.2, Omicron BA.2.76, Omicron BA.3, Omicron BA.4, and Omicron BA.5. The 87 sequences manifested as an independent evolutionary cluster, forming a branch with Omicron BA.2 and a sub-branch with Omicron BA.2.76. The evolution of the SARS-CoV-2 genome in these 87 Omicron BA.2.76 sequences from Jining City consistently corresponds to the Pangolin classification. The resultant evolutionary tree delineates two distinctive branches, implying the plausible existence of two principal transmission chains within the Omicron BA.2.76 lineage in Jining City (Fig. 2).

### Genetic mutation analysis

In comparison to the Wuhan-Hu-1 reference, the 87 Omicron BA.2.76 sequences from Jining City exhibit variations at 77–84 nucleotide positions and 27 nucleotide deletions. Internally, among the 87 sequences, there are 0–9 nucleotide differences. Cumulatively, these sequences display 105 nucleotide mutations, with four occurring in non-coding regions—specifically, in the 5’UTR, ORF8 translation initiation region, N translation initiation region, and 3’UTR non-coding region. Among the nucleotide variations, 101 are located within coding regions, with the ORF1ab and S genes experiencing the most nucleotide changes. Within these, the ORF1ab gene exhibited 48 nucleotide mutations and 9 nucleotide deletions, while the S gene showed 34 nucleotide mutations and 9 nucleotide deletions. Furthermore, within the analysis of the 87 Omicron BA.2.76 sequences from Jining City, a suite of mutations—G777A, C884T, C1108T, C1973T, C2197T, A2869G, T4240C, C4321T, C7279T, T7302C, C7528T, C7735T, A9055G, A10323G, C10965T, G11083T, C11173T, C14697T, G14829T, T15232G, C15738T, G16187A, G16188T, C16293T, C17024T, G18157T, G19009T, G19684T, C20132T, C22120T, A22491G, A23173G, C23202T, C26408T, A27458G, C27972T, A28271T, G29692A—emerged as distinctive to this specific outbreak, appearing less commonly across other sequences of the virus. Among these, the A10323G and G14829T mutations were notably prevalent, with the A10323G mutation present in 40.23% (35 of 87) and the G14829T mutation present in 42.53% (37 of 87) of the sequences analyzed. These variations suggest that individuals harboring these mutations may have played a significant role in forming crucial transmission chains during the current outbreak (Table 1).

**Table 1:** Compares genetic variations in the Omicron BA.2.76 sequence between Jining City and Wuhan-Hu-1

Gene	Protein	Gene mutation site	Protein variation site
5’UTR	-	C241T(87/87)	-
*ORF1ab*	NSP1	T670G(87/87), G777A(1/87), C884T(6/87), C1108T(87/87), C1973T(1/87), C2197T(1/87), C2790T(87/87), A2869G(1/87), C3037T(87/87), G4184A(87/87), T4240C(1/87), C4321T(87/87), C7279T(3/87), T7302C(2/87), C7528T(87/87), C7735T(1/87), A9055G(87/87), C9344T(87/87), A9424G(87/87), C9534T(87/87), C9866T(87/87), C10029T(87/87), C10198T(87/87), A10323G(35/87), G10447A(87/87), A10449A(87/87), C10965T(1/87), G11083T(1/87), C11173T(87/87), TCTGGTTTT11288-11296 missing, C12880T(87/87), C14408T(87/87), C14697T(2/87), G14829T(37/87), T15232G(1/87), C15714T(87/87), C15738T(1/87), G16187A(3/87), G16188T(3/87), C16293T(3/87), C17024T(87/87), C17410T(87/87), G18157T(1/87), A18163G(87/87), G19009T(87/87), G19684T(87/87), C19955T(87/87), A20055G(87/87), C20132T(3/87)	S135R(87/87), R171H(1/87)
	NSP2		R27C(6/87)
	PLpro		T24I(87/87), G489S(87/87), I1528T(2/87)
	NSP4		L264F(87/87), T327I(87/87), L438F(87/87), T492I(87/87)
	3CLpro		K90R(35/87), P132H(87/87), T304I(1/87)
	NSP6		L37F(1/87), SGF106-108 missing
	RdRp		P323L(87/87), M463I(37/87), W598G(1/87), W916Y(3/87)
	helicase		S263F(87/87), R392C(87/87)
	NSP14		V40F(1/87), I42V(87/87), D324Y(87/87)
	NSP15		V22L(87/87), T112I(87/87), A171V(3/87)
*S*	S	C21618T(87/87), TACCCCCTG2633-21641missing, G21987A(87/87), C22120T(3/87), T22200G(87/87), T22304A(87/87), A22491G(2/87), G22578A(87/87), G22599C(87/87), C22674T(87/87), T22679C(87/87), C22686T(87/87), A22688G(87/87), G22775A(87/87), A22786C(87/87), G22813T(87/87), T22882G(87/87), G22992A(87/87), C22995A(87/87), A23013C(87/87), A23040G(87/87), A23055G(87/87), A23063T(87/87), T23075C(87/87), A23173G(1/87), C23202T(1/87), A23403G(87/87), C23525T(87/87), T23599T(87/87), C23604A(87/87), C23854A(87/87), G23948T(87/87), A24424T(87/87), T24469A(87/87), C25000T(87/87)	T19I(87/87), L24S(87/87), PPA25-27 missing(87/87), G142D(87/87), V213G(87/87), Y248N(87/87), K310R(2/87), G339D(87/87), R346T(87/87), S371F(87/87), S373P(87/87), S375F(87/87), T376A(87/87), D405N(87/87), R408S(87/87), K417N(87/87), N440K(87/87), S477N(87/87), T478K(87/87), E484A(87/87), Q493R(87/87), Q498R(87/87), N501Y(87/87), Y505H(87/87), T547I(1/87), D614G(87/87), H655Y(87/87), N679K(87/87), P681H(87/87), N764K(87/87), D796Y(87/87), Q954H(87/87), N969K(87/87)
*ORF3a*	ORF3a	C25416T(87/87), C25584T(87/87), C26060T(87/87)	T223I(87/87)
*E*	E	C26270T(87/87), C26408T(1/87)	E9I(87/87), S55F(1/87)
*M*	M	C26577G(87/87), G26709A(87/87), C26858T(87/87)	Q19E(87/87), A63T(87/87),
*ORF6*	ORF6	A27259C(87/87), G27382C(87/87), A37383T(87/87), T27384C(87/87)	D61L
*ORF7a*	ORF7a	A27458G(1/87)	E22G(1/87)
*ORF8* TIR	-	C27807T(87/87),	-
*ORF8*	ORF8	C27972T(87/87)	Q27stop (87/87)
N TIR	-	A28271T(87/87),	-
*N*	N	C28311T(87/87), GAGAACGCA28362-28370 deletion, G28881A(87/87), G28882A(87/87), G28883C(87/87), A29510C(87/87)	P13L(87/87), ERS31-33 deletion (87/87), R203K(87/87), G204R(87/87), S413R(87/87),
3’UTR		G29692A(1/87)	-

TIR: translation initiation region

### Non-coding region compilation analysis

**Fig. 3:**
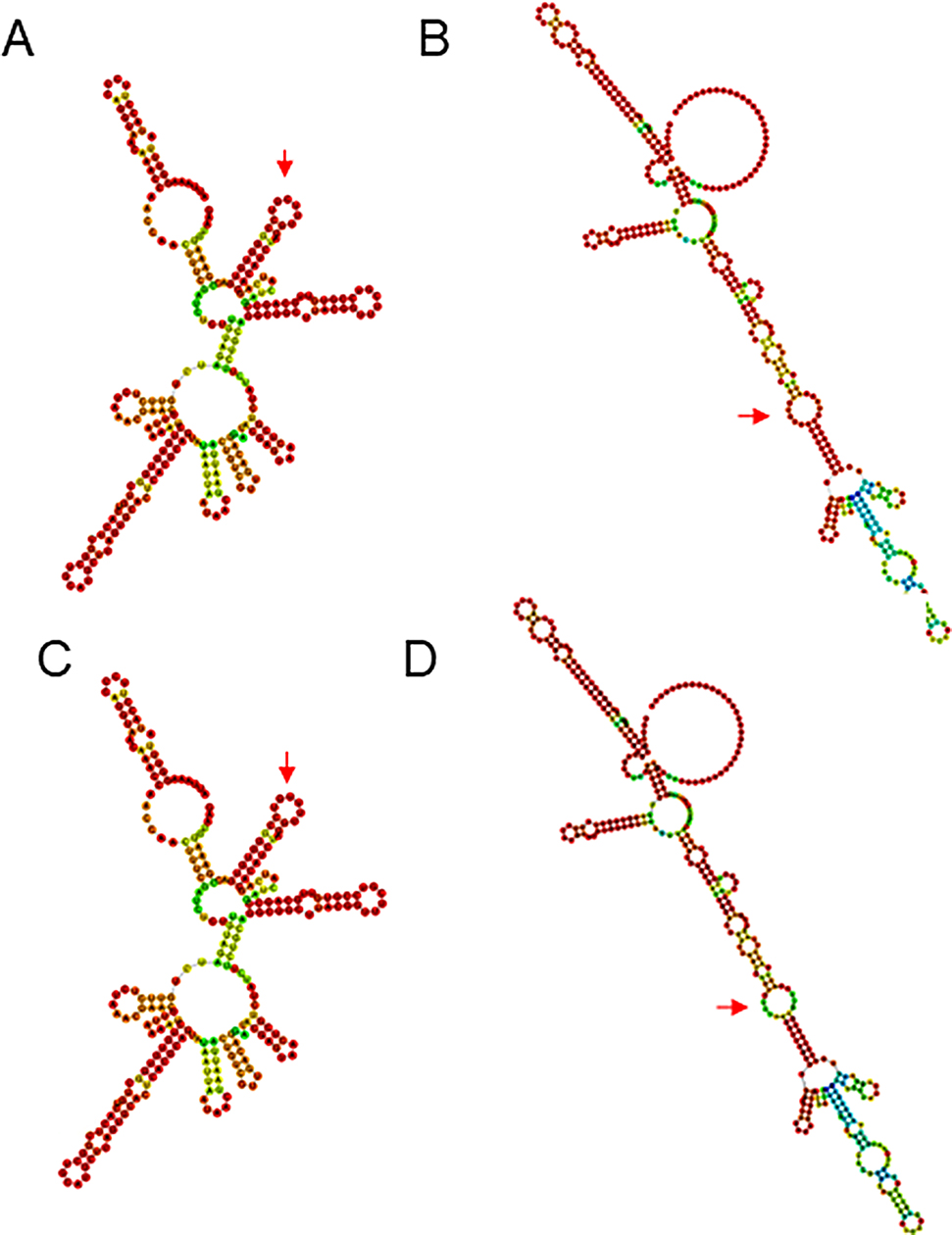
.

In the 87 Omicron BA.2.76 sequences from Jining City, a C241T mutation was observed at the 241 nucleotide position within the stem-loop structure of the 5’UTR SL5B [8]. Additionally, one sequence exhibited a G29692A mutation in the hypervariable region (HVR) of the 3’UTR [8]. Utilizing RNAfold software, secondary structures for both the 5’UTR and 3’UTR were constructed. The C241T mutation in the 5’UTR and the G29692A mutation in the 3’UTR were identified within loop structures. Both mutation sites are not located in the stem structures of the 5’UTR and 3’UTR, not affecting the nucleotide pairing within these regions, thereby exerting minimal impact on the secondary structures of both areas (Fig. 3).

**Fig. 4:**
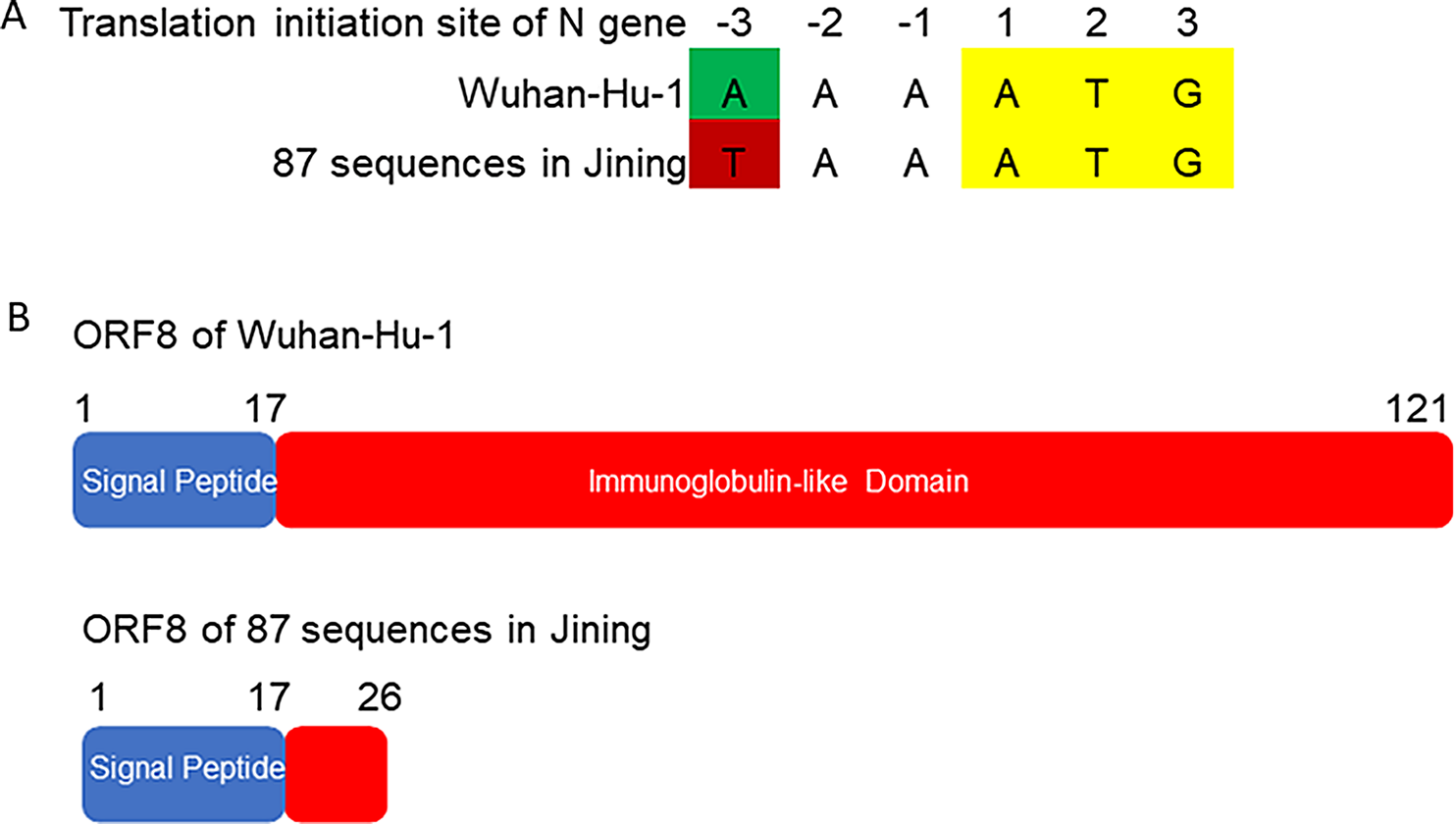
.

Within the 87 Omicron BA.2.76 sequences from Jining City, a consistent A28271T mutation was identified in the N translation initiation region. The nucleotide at position 28,271, situated at -3 in the N translation initiation region, underwent a change from A to T due to the A28271T mutation (Fig. 4A). Upon analysis of SARS-CoV-2 variant sequences downloaded from GISAID, it was observed that the A28271T mutation is present in SARS-CoV-2 Alpha, Delta, and Omicron variants.

### Coding region compilation analysis

#### General overview

In the 87 Omicron BA.2.76 sequences from Jining City, a total of 69 amino acid residue mutations were observed, along with 9 amino acid deletions at three locations, and one termination mutation. Amino acid mutations occurred in 18 proteins, with the S protein exhibiting the highest mutation frequency—32 amino acid positions were altered, and one location had a deletion of three amino acid residues. Following S protein, the N protein had 5 amino acid mutations, including one location with a deletion of three amino acids (Table 1).

#### S protein mutation analysis

The S protein, a receptor-binding protein of SARS-CoV-2 [9], experienced 32 amino acid residue mutations in the 87 Omicron BA.2.76 sequences from Jining City. Among these, 28 mutations were located in the S1 region, and 4 in the S2 region. Notably, 17 mutations occurred in the receptor-binding region, including G339D, R346T, S371F, S373P, S375F, T376A, D405N, R408S, K417N, N440K, S477N, T478K, E484A, Q493R, Q498R, N501Y, and Y505H.

#### ORF8 protein termination mutation analysis

The open reading frame 8 (ORF8) in all 87 Omicron BA.2.76 sequences from Jining City exhibited a C27972T mutation, resulting in the conversion of the nucleotide coding for the 27th amino acid in the ORF8 protein from CAA to the stop codon TAA. Consequently, the encoded protein underwent a termination mutation, generating a truncated ORF8 protein named ORF8 Q27 Termination Protein. ORF8 Q27 Termination Protein comprises only the first 26 amino acids of the ORF8 protein, including the complete signal peptide’s initial 17 amino acids and the first 9 amino acids of the N-terminal immunoglobulin-like domain (Fig. 4B).

#### N-Glycosylation analysis

Utilizing NetNGlyc-1.0 software to analyze protein N-glycosylation sites, the 87 Omicron BA.2.76 sequences from Jining City exhibited the T24I mutation in the papain-like protease (Plpro), resulting in the alteration of NIT22-24 to NII22-24 and the loss of the N22 glycosylation site. Additionally, the S protein in the 87 sequences had the T19I mutation, leading to the modification of NLT17-19 to NLI17-19 and the loss of the N17 glycosylation site. Furthermore, the Y248N mutation in the S protein resulted in the transformation of YLT248-250 to NLT248-250, introducing an additional N248 glycosylation site (Table 2).

**Table 2:** Analysis of variations in N-glycosylation and phosphorylation sites in the 87 sequences of Omicron BA.2.76 in Jining City, compared with Wuhan-Hu-1

Protein	Amino acid positions of Wuhan-Hu-1	Amino acid positions in the Jining City sequence	Number of mutations in the 87 sequences of Jining City	Changes in N-glycosylation sites or phosphorylation sites
Plpro	NIT22-24	NII22-24	87	Loss of N22 glycosylation site
S	NLT17-19	NLI17-19	87	Loss of N17 glycosylation site
S	YLT248-250	NLT248-250	87	Addition of N248 glycosylation site
NSP1	S135	R135	87	Loss of S135 phosphorylation site
Plpro	T24	I24	87	Loss of T24 phosphorylation site
Plpro	G489	S489	87	Addition of S489 phosphorylation site
NSP6	S106	-	87	Loss of S263 phosphorylation site
helicase	S263	F263	87	Loss of T24 phosphorylation site
S	T19	I19	87	Loss of T19 phosphorylation site
S	S375	F375	87	Loss of S375 phosphorylation site
S	T376	A376	87	Loss of T376 phosphorylation site
S	S477	N477	87	Loss of S477 phosphorylation site
S	T478	K478	87	Loss of T478 phosphorylation site
S	T547	I547	1	Loss of T547 phosphorylation site
S	L24	S24	87	Addition of S24 phosphorylation site
S	R408	S408	87	Addition of S408 phosphorylation site
S	H655	Y655	87	Addition of Y655 phosphorylation site
ORF3a	T223	I223	87	Loss of T223 phosphorylation site
E	S55	F55	1	Loss of S55 phosphorylation site
N	S33	-	87	Loss of S33 phosphorylation site
N	S413	T413	87	Loss of S413 phosphorylation site

Note: “-” represents a missing mutation

#### Phosphorylation analysis

Phosphorylation site analysis using NetPhos-3.1 revealed several modifications in the 87 Omicron BA.2.76 sequences from Jining City. Notably, the non-structural protein 1 (NSP1) lost the S135 phosphorylation site, and the papain-like protease (Plpro) lost the T24 phosphorylation site while gaining an S489 phosphorylation site. The NSP6 protein lost the S106 phosphorylation site, the helicase lost the S263 phosphorylation site, and the S protein lost phosphorylation sites at T19, S375, T376, S477, T478, and T547. Conversely, the S protein gained phosphorylation sites at S24, S408, and Y655. Additionally, the ORF3a protein lost the T223 phosphorylation site, and the E protein lost the S55 phosphorylation site. The N protein lost phosphorylation sites at S33 and S413 (Table 2).

## Discussion

The Omicron variant, characterized by increased transmissibility and a faster transmission rate, has emerged as the predominant strain of SARS-CoV-2, reshaping the landscape of the pandemic [5–7, 10]. This study’s sequences, originating from a COVID-19 outbreak caused by the Omicron BA.2.76 variant in Jining City, Shandong Province, China, exhibit high homogeneity and form an independent cluster within the evolutionary tree. The comparison with the Wuhan-Hu-1 strain revealed 105 nucleotide mutations and 27 deletions, indicating the variant’s significant genetic evolution. These findings include mutations in non-coding regions such as C241T in the 5’UTR and G29692A in the 3’UTR, whose impacts on the virus’s replication and translation mechanisms warrant further investigation.

The − 3 nucleotide position at the eukaryotic translation initiation site is pivotal for translation efficiency, where − 3 A interacts with eukaryotic initiation factor 2α (eIF2α), enhancing the recognition of the translation initiation site (TIS) and protein synthesis [11–14]. Given that viruses utilize the host’s translational machinery for protein synthesis, the A28271T mutation, which changes − 3 A to -3T in the N gene’s translation initiation region, could impair the translation efficiency of the N gene, potentially leading to diminished N protein levels in host cells [15–18]. The N protein, crucial for SARS-CoV-2 structure, significantly influences cytokine storm induction through the promotion of inflammatory factor production, exacerbating pneumonia in those infected [19–22]. Thus, a reduction in N protein expression might attenuate the inflammatory response, potentially moderating the severity of pneumonia.

Notably, the S protein mutations identified, including D614G, S477N, E484K, N501Y, T478K, and Q498R, suggest enhanced transmissibility and immune evasion, posing challenges to current vaccine efficacy and necessitating ongoing surveillance and research to adapt vaccine formulations [23–25, 9, 26–29]. Additionally, the ORF8 protein, by inhibiting the host’s type I interferon (IFN-1) signaling pathway, evading host immune clearance, and inducing cytokine storm, significantly contributing to fibrosis and coagulation dysfunction. The significance of fibrosis, in particular, cannot be overstated due to its severe implications for human health [30–44]. The Q27 premature termination mutation in ORF8, found across all sequences, might reduce the virus’s virulence, affecting its ability to induce lung fibrosis and coagulation dysfunction, which are critical factors in COVID-19 severity [45–47].

Post-translational modifications (PTMs), such as glycosylation and phosphorylation, are vital for the replication, assembly, release, and elicitation of host immune responses to viruses, including SARS-CoV-2 [48–53]. Specifically, the SARS-CoV-2 S protein, which has 22 glycosylation sites, undergoes modifications affecting its structure, receptor binding, and interference with host immune responses [53, 54]. Notably, in the 87 sequences studied, the alteration of glycosylation sites, including the loss of N17 and the addition of N248, prompts further examination of their roles in viral pathogenicity Additionally, the extensive presence of over 70 phosphorylation sites across SARS-CoV-2 proteins, implicated in essential viral functions, is noteworthy [54, 55]. The identification of changes in 18 phosphorylation sites across eight proteins in this study hints at significant alterations in their roles, necessitating further exploration of these modifications’ effects on the virus’s behavior.

Recent findings underscore the Omicron variant’s increased transmissibility, yet its impact on disease severity varies markedly across different populations [56]. This variation presents a spectrum of clinical outcomes, suggesting less severe symptoms compared to earlier variants. Such variability, influenced by both strain-specific characteristics and vaccination status, underscores the ongoing need for vigilance and targeted research [57, 58]. Our focused examination of Omicron variant BA.2.76 in Jining City sheds light on this variant’s behavior, highlighting the critical role of localized data in understanding the pandemic’s evolving dynamics.

Moreover, the response to Omicron’s spread has necessitated a reassessment of vaccine efficacy. Preliminary analyses indicate a diminished neutralizing response, prompting a recalibration of vaccination strategies, such as booster doses and potential vaccine formula adjustments, crucial for sustaining global vaccination efforts [59]. The reliability of diagnostic tests in the face of Omicron’s emergence has sparked rigorous evaluations, with current evidence supporting the continued effectiveness of most PCR and antigen tests [60]. This ensures the integrity of diagnostic protocols, essential for effective pandemic management. Simultaneously, the global initiative to sequence the SARS-CoV-2 genome has been pivotal in tracking the variant’s spread and evolution, providing invaluable insights into its global distribution. This sequencing effort, by analyzing data from diverse regions, aids in identifying mutation patterns and transmission dynamics, informing public health strategies. Additionally, the advent of Omicron raises significant questions regarding the current therapeutic options’ efficacy, including monoclonal antibodies and antiviral drugs. The nuanced impact observed necessitates continuous research into therapeutic strategies, highlighting the importance of adapting treatment approaches to effectively combat the challenges presented by emerging viral strains.

In conclusion, this study reports 87 sequences of the Omicron BA.2.76 variant, offering significant insights into the genomic landscape of the Omicron variant worldwide. These results enhance our grasp of SARS-CoV-2’s evolutionary dynamics and offer crucial data for assessing shifts in the virus’s pathogenicity.

### Electronic supplementary material

Below is the link to the electronic supplementary material.

Supplementary Material 1

## Data Availability

Data is provided within the supplementary information files.
